# Supplementary material for: The causal role of circulating inflammatory markers in osteoporosis: a bidirectional Mendelian randomized study
Source: Front Immunol. 2024 Jul 18;15:1412298. doi: 10.3389/fimmu.2024.1412298 (PMC11291241; doi:10.3389/fimmu.2024.1412298)
Supplement: Supplementary file 1 [file DataSheet_1.zip › supplementary material/CIM-OSTEOPOROSIS /GCST90274808.csv_leave_one_out.pdf]

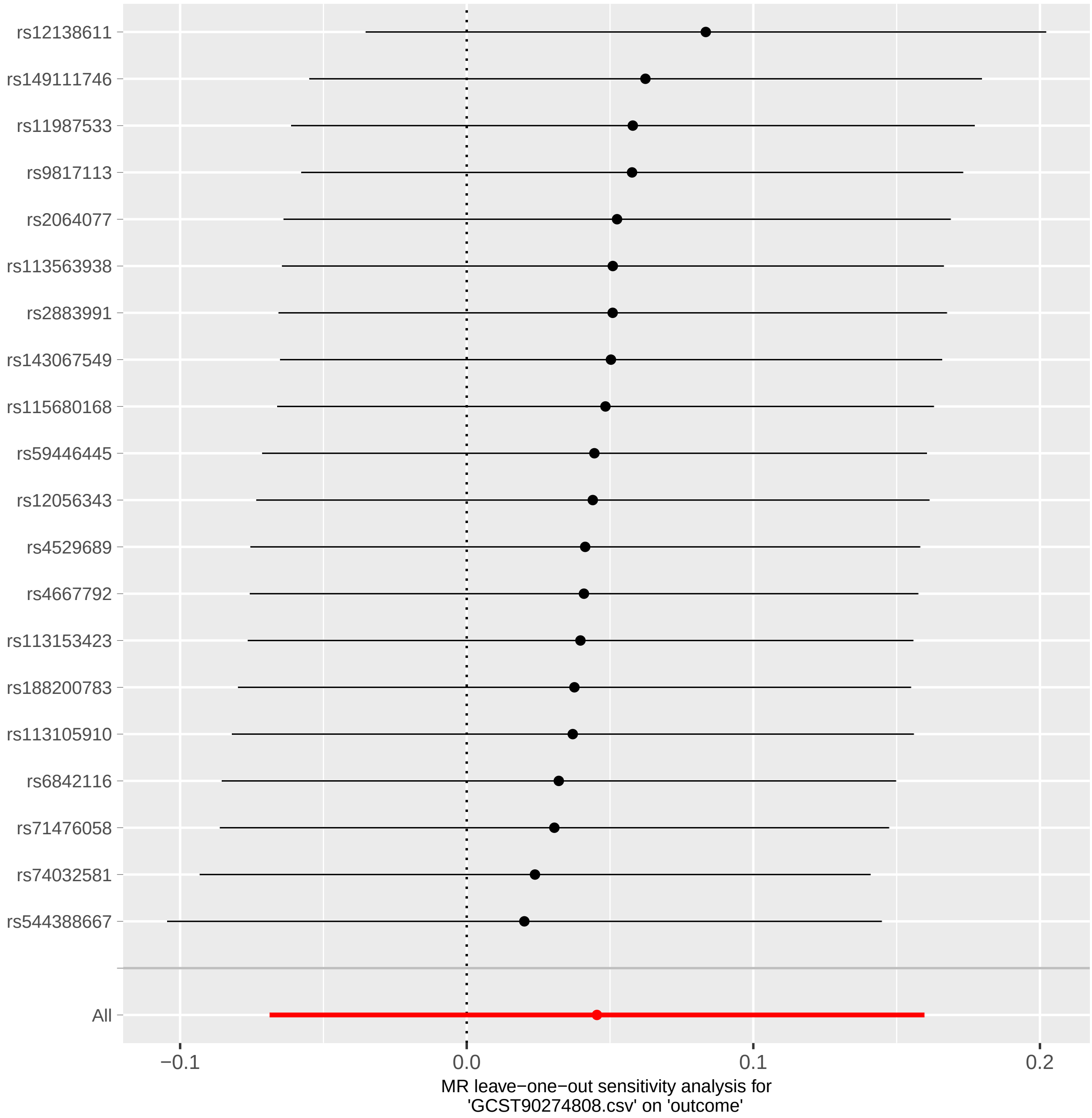
rs12138611
rs149111746
rs11987533
rs9817113
rs2064077
rs113563938
rs2883991
rs143067549
rs115680168
rs59446445
rs12056343
rs4529689
rs4667792
rs113153423
rs188200783
rs113105910
rs6842116
rs71476058
rs74032581
rs544388667
All
−0.1
0.0
0.1
0.2
MR leave−one−out sensitivity analysis for
'GCST90274808.csv' on 'outcome'
